# Strategies to Improve Hydrolysis Efficiency of Fish Skin Collagen: Study on ACE Inhibitory Activity and Fibroblast Proliferation Activity

**DOI:** 10.3390/foods13233869

**Published:** 2024-11-29

**Authors:** Cuihua Chang, Yuzhou Ma, Yanjun Yang, Yujie Su, Luping Gu, Junhua Li

**Affiliations:** 1 State Key Laboratory of Food Science and Resources, Jiangnan University, Wuxi 214122, China; chang.cuihua@jiangnan.edu.cn (C.C.); 1012220224@stu.jiangnan.edu.cn (Y.M.); yangyj@jiangnan.edu.cn (Y.Y.); suyujie@jiangnan.edu.cn (Y.S.); guluping@jiangnan.edu.cn (L.G.); 2 School of Food Science and Technology, Jiangnan University, Wuxi 214122, China

**Keywords:** fish skin collagen, dual enzyme, triple enzyme, ACE inhibitory activity, fibroblast proliferation activity

## Abstract

Collagen peptides play a crucial role in promoting skin elasticity and enhancing joint health, with potential functions to be explored. Enzyme hydrolysis is crucial for the molecular weight and sequence of peptides, influencing the bio-activity. In this study, the angiotensin-converting enzyme (ACE) inhibitory activity and fibroblast proliferation activity of differentially molecular weight peptides derived from dual- or triple-enzyme hydrolysis were compared. Ultrafiltration membrane filtration was used to separate the hydrolyzed prepared collagen peptides into two components based on the molecular size. The results showed that the low-molecular-weight peptide fraction containing peptides with P at the C-terminal, such as KP, RP, and POGP, exhibited high ACE inhibitory activity. The low-molecular-weight peptide fraction obtained through triple-enzyme hydrolysis incorporating ginger protease exhibited the highest ACE inhibitory activity, with an IC50 3.1 mg/mL. In addition, the triple-enzyme hydrolyzed collagen peptides passing across membranes displayed higher migration rates and enhanced collagen synthesis capabilities, containing peptide sequences, such as POGP, POGA, and LPO, potentially promoting fibroblast proliferation. The results would provide practical guidance for the production of collagen peptides with high ACE inhibitory activity and fibroblast proliferation activity, in terms of enzyme processing and highly active peptide separation.

## 1. Introduction

Collagen constitutes approximately 30% of the total protein content in animals and is ubiquitous in skin, tendons, bones, cartilage, and other tissues and organs, playing a pivotal role in maintaining their structural integrity and functionality [1]. Due to its unique structure, low allergenicity, and excellent biocompatibility, collagen has extensive applications in medical biomaterials, particularly in wound hemostasis and tissue repair. Additionally, as a natural biological material, collagen is widely utilized in cosmetics, food, and health supplements [2]. Notably, over 66% of processing waste from livestock and aquaculture industries, including fins, heads, skins, and viscera, are currently disposed of through composting, incineration, or landfilling, despite containing high concentrations of collagen that could be converted into valuable products to enhance byproduct utilization [3].

Nowadays, with the increasing pursuit of a high-quality life, bioactive peptides (BPs) have emerged as prominent functional ingredients [4]. Extracted from food proteins, BPs exhibit an array of beneficial properties, with current research focusing on their potential as nutritional supplements and functional food components [5]. Since 1993, collagen-derived bioactive peptides (BPCs) have been classified by the US Food and Drug Administration as “Generally Recognized as Safe” (GRAS) [6]. Beyond their high safety profile upon oral ingestion, BPCs boast exceptional water solubility, rendering them ideal for functional food formulations, such as high-protein beverages and functional foods [7]. Notably, collagen peptides exhibit enhanced bioavailability compared to collagen and gelatin, facilitating their absorption and conferring ACE inhibitory and antioxidant activities, thereby positioning them as prime candidates for dietary protein sources and food ingredients [8].

The enzyme type plays a typical role in the generation of functional peptides, determining the peptide molecular weight distribution and sequence. As reported, the bone collagen hydrolysate prepared with alkaline protease yielded the highest ACE inhibitory activity [9]. The molecular weight of hydrolysates has a strong effect on the ACE inhibition activity of collagen peptide, with low-molecular-weight peptides exhibiting further enhanced activity after ultrafiltration fractionation. The isolation and identification of ACE inhibitory peptides from collagen have garnered significant research interest. For instance, a novel ACE inhibitory peptide (GHVGAAGS) was purified from commercial fish collagen hydrolysates, and another (GPPGADGQAGAK) was isolated from sturgeon skin hydrolysates [10]. For small peptides, especially collagen triple peptides (CTPs), the high hydrophobicity of the C-terminal amino acids in CTP is intimately associated with ACE (angiotensin-converting enzyme) inhibitory activity, substantially enhancing the binding capacity between peptides and ACE [11]. Peptides (such as Hyp-Pro-Gly, Hyp-Pro, and Ala-Hyp) can be transported into the bloodstream and reach target tissues (skin, bone, or joints) following oral administration [12]. In addition, smaller peptides exhibit resistance to degradation by gastrointestinal digestive enzymes and plasma enzymes, facilitating their superior absorption. Caco-2 cell absorption experiments on collagen hydrolysates also revealed a molecular weight-dependent permeability of various peptide segments, with smaller molecular weight peptides preferentially transported by peptide transporters [13].

However, current research on collagen peptides faces several challenges. Firstly, most studies concentrate on isolating functional peptide sequences from bulk enzymatic hydrolysis, neglecting the development of efficient preparation methods for small molecule peptides (less than 1 kDa), which provides limited guidance for industrial processing. Secondly, although some studies suggest a correlation between peptide activity and specific sequences (such as ACE inhibitors often ending in proline), a targeted hydrolysis method to increase the abundance of these special peptide sequences remains unknown. To overcome these issues, this study utilized dual- or triple-enzyme hydrolysis of fish skin collagen to produce small peptides with overall molecular weights below 1 kDa. The study evaluated the hydrolysis efficiency of alkaline, papain, and ginger proteases, comparing the peptide sequence under dual- and triple-enzyme hydrolysis. The hydrolysates were then separated into two components using an ultrafiltration membrane with a cut-off molecular weight of 2000 Da. One component contained more large molecular weight peptide sequences, and the other component contained more small molecular weight peptide sequences. The ACE inhibitory activity and fibroblast proliferation activity of these separated components were compared to clarify the relationship between complex enzymatic hydrolysis processes, collagen peptide sequence distributions, and their physiological activities. This research work is meaningful as it provides data support and practical guidance for the industrial production of targeted collagen peptides with specific physiological activities.

## 2. Materials and Methods

### 2.1. Materials and Chemicals

Fish skin gelatin was purchased from Wuhan Lanabai Pharmaceutical Chemical Co., Ltd. (Wuhan, China). Alcalase (1 × 10^5^ U/g) was purchased from Angel Yeast Co., Ltd. (Yichang, China). Papain (2 × 10^5^ U/g) was purchased from Nanning Pangbo Biological Engineering Co., Ltd. (Nanning, China). Ginger protease (2 × 10^5^ U/mL) was purchased from Royal DSM Co., Ltd. (Auckland, The Netherlands). Gly-Pro-Hyp standard was purchased from Shanghai Qiangyao Biological Technology Co., Ltd. (Qingdao, China). Angiotensin-converting enzyme and N-Hippuryl-His-Leu hydrate were purchased from Sigma-Aldrich Trading Co., Ltd. (Shanghai, China). Mouse embryonic fibroblast cells (NIH-3T3) were provided by Stem Cell Bank, Chinese Academy of Sciences. CCK-8 kits were purchased from Beyotime Biotechnology Co., Ltd. (Shanghai, China). All the other reagents used were analytical grade.

### 2.2. Collagen Hydrolysates Preparation

The fish skin collagen solution (5%, *w*/*v*) was treated by alcalase (3000 U/g protein) at pH 10.0 for 2 h under 55 °C, papain (3000 U/g protein) at pH 6.0 for 2 h under 55 °C, or natural protease (3000 U/g protein) at pH 7.0 for 2 h under 55 °C. The samples treated by single alcalase, papain, or natural protease were named AA, PP, and NN, respectively. These samples were hydrolyzed under the optimum pH value.

The sample treated by the combination of alcalase and papain was named AP or PA, depending on the enzyme addition order. For the AP sample, the collagen protein solution was adjusted to pH 10.0 before adding alcalase. After 2 h of reaction, the pH was adjusted to 6.0, and papain was added for further hydrolysis. For the PA sample, the collagen protein solution was adjusted to pH 6.0 before adding papain. After 2 h of reaction, the pH was adjusted to 10.0, and alcalase was added for further hydrolysis.

The sample treated by the combination of alcalase and natural protease was named AN or NA, depending on the enzyme addition order. For the AN sample, the collagen protein solution was adjusted to pH 10.0 before adding alcalase. After 2 h of reaction, the pH was adjusted to 7.0, and natural protease was added for further hydrolysis. For the NA sample, the collagen protein solution was adjusted to pH 7.0 before adding natural protease. After 2 h of reaction, the pH was adjusted to 10.0, and alcalase was added for further hydrolysis.

The sample treated by the combination of papain and natural protease was named PN or NP, depending on the enzyme addition order. For the PN sample, the collagen protein solution was adjusted to pH 6.0 before adding papain. After 2 h of reaction, the pH was adjusted to 7.0, and natural protease was added for further hydrolysis. For the NA sample, the collagen protein solution was adjusted to pH 7.0 before adding natural protease. After 2 h of reaction, the pH was adjusted to 6.0, and papain was added for further hydrolysis.

To investigate the effect of the triple enzyme on the hydrolysis efficiency, ginger protease was used in the AP sample. After the hydrolysis of alcalase and papain, ginger protease (3000 U/g protein) was added, reacting at pH 4.5 for 2 h under 55 °C, recorded as APG.

### 2.3. Hydrolysis Rate

The degree of hydrolysis (DH) of the sample was determined using the o-phthalaldehyde (OPA) method. The specific experimental procedure was as follows: Two solutions, A and B, were prepared. Solution A involved completely dissolving 80 mg of OPA in 2 mL of anhydrous ethanol under light-protected conditions. Solution B was prepared by dissolving 1.91 g of sodium tetraborate, 0.1 g of sodium dodecyl sulfate, and 88 mg of 1,4-dithiothreitol together in 50 mL of deionized water. Subsequently, solutions A and B were mixed and diluted with deionized water to a total volume of 100 mL. Then, 0.4 mL of the sample solution was thoroughly mixed with 3 mL of the OPA solution to obtain the solution to be tested. After allowing the solution to stand for 2 min under light-protected conditions, its absorbance at 340 nm was measured using a UV–Vis spectrophotometer.

The DH was calculated using formulas (1) and (2). Deionized water was used as the blank control, and 0.1 mg/mL L-serine (0.9516 meqv/L) was used as the standard control.

(1) $$\mathrm{Serine-N}H_{2}=\frac{{\mathrm{OD}}_{\mathrm{sample}}-{\mathrm{OD}}_{\mathrm{blank}}}{{\mathrm{OD}}_{\mathrm{standard}}-{\mathrm{OD}}_{\mathrm{blank}}}\times 0.9516\mathrm{meqv}/L\times \frac{0.1\times 100}{X\times P}$$

In Equation (1), OD_sample_ represents the absorbance of the sample solution; OD_blank_ is the absorbance of deionized water as the blank control; OD_standard_ is the absorbance of the 0.1 mg/mL L-serine solution as the standard control; X is the mass of the sample (g); and P is the concentration of protein in the sample (%).

(2) $$\mathrm{DH}\left(\%\right)=\frac{h}{h_{\mathrm{tot}}}=\frac{\mathrm{Serine-N}H_{2}-\beta }{\alpha \times h_{\mathrm{tot}}}$$

In Equation (2), h represents the number of peptide bonds broken per unit weight of the sample (mmol/g); h_tot_ is the total number of peptide bonds per unit weight of the sample (mmol/g), which was derived from Equation (1); for gelatin, α is 0.796, β is 0.457, and h_tot_ is 11.1.

### 2.4. Membrane Separation

The collagen peptides prepared by dual or triple enzymes were filtrated using an ultrafiltration membrane with a molecular weight cut-off (MWCO) of 2000 Da (bought from Toray, Japan). The membrane captured a peptide sequence with a molecular size larger than 2000 Da. After 80% of the liquid passed through the membrane, an equal volume of water was added, and 80% was filtered again. All the retained liquid and the permeate were collected and dried using a freezer, for further analysis. For the dual-enzyme hydrolyzed sample, the retained phase was referred to as AP-1 (containing more large molecular weight collagen peptides), and the permeate was referred to as AP-2 (containing more small molecular weight collagen peptides). For the triple-enzyme hydrolyzed sample, the retained phase was referred to as APG-1 (containing more large molecular weight collagen peptides), and the permeate was referred to as APG-2 (containing more small molecular weight collagen peptides).

### 2.5. Molecular Weight Distribution

The determination of the molecular weight distribution of collagen peptides was performed using a TSK G2000SW (300 × 7.5 mm) column on an HPLC. The operation parameters were as follows: mobile phase composition: 39.95% water, 60% acetonitrile, 0.05% trichloroacetic acid (TCA); flow rate: 1 mL/min; detection wavelength: 214 nm; injection volume: 10 μL. A molecular weight calibration curve was prepared using the average elution volume of the following standards: cytochrome C (12,384 Da), insulin from bovine pancreas (5733.49 Da), bacitracin (1422.69 Da), L-glutathione reduced (307.32 Da), and glycine (75.07 Da).

### 2.6. Peptide Sequences

An HPLC−ESI-MS/MS (MALDI SYNAPT MS, Waters, American) was used to identify the sequences of characteristic collagen peptide. The instrument parameters were set based on our previous experiments. Then, the software MassLynx V4.1 was used to analyze the identified peptide sequences. The analysis of collagen peptides required 100 mg of the freeze-dried sample diluted in 20 mL deionized water to a final concentration of 5 mg/mL. After filtration by a 0.45 μm membrane, the permeate was analyzed by ICP−MS-MS (NexION 350D, PerkinElmer, American) combined with HPLC online. The instrument parameters were as follows: column: ESA III anion-exchange column (250 mm × 4.6 mm); mobile phase: 25 mmol/L ammonium citrate solution, pH 3.1; flow rate: 1 mL/min; injection volume: 20 μL.

### 2.7. ACE Inhibitory Activity

ACE inhibitory activity was determined as follows: first, 100 μL of sodium borate buffer (0.1 M) and 20 μL of collagen peptides were mixed with an aliquot (200 μL) of hippuryl-histidyl-leucine (Sigma-Aldrich) solution (5 mM hippuryl-histidyl-leucine in 0.1 M sodium borate buffer containing 0.3 M NaCl, pH 8.3) and incubated at 37 °C for 30 min. The reaction was activated by adding 40 μL of ACE (Sigma-Aldrich) solution (0.1 U/mL, pH 8.3) and incubated in a water bath (37 °C) for 30 min. Then, 200 μL of 1 M HCl was added to terminate the reaction, after which 1.2 mL of ethyl acetate was added to the mixture to extract the released hippuric acid. After the samples were vigorously stirred for 10 s, they were centrifuged at 5000× *g* for 5 min; then, 0.8 mL of the organic phase was transferred to a fresh test tube. The ethyl acetate was evaporated to dryness in a drying oven. The residue was dissolved in 1.0 mL of deionized water, and the absorbance was measured using a spectrophotometer at 228 nm. The following equation was used to calculate the ACE inhibitory activity, where A represents the absorbance with ACE but without the sample, B denotes the absorbance in the presence of ACE and the sample, while C represents the absorbance without ACE and the sample. The ACE inhibition rate was calculated as follows:

(3) $$\mathrm{Inhibition}(\%)=\frac{{\mathrm{OD}}_{A}-{\mathrm{OD}}_{B}}{{\mathrm{OD}}_{A}-{\mathrm{OD}}_{C}}\times 100\%.$$

In the formula, OD_A_ represents the optical density when no inhibitor is present, OD_B_ represents the optical density when both the inhibitor and enzyme are present, and OD_C_ represents the optical density when neither the inhibitor nor enzyme is present. The concentration of the inhibitor that achieves 50% inhibition of ACE is defined as the half maximal inhibitory concentration (IC_50_).

### 2.8. Cell Culture

NIH-3T3 fibroblasts were maintained in Dulbecco’s modified Eagle’s medium (DMEM) containing 10% fetal calf serum (FCS) and 1% penicillin/streptomycin until confluence, hereinafter referred to as the complete medium. The cytotoxicity of NIH-3T3 preadipocytes was measured by the CCK-8 kit. NIH-3T3 fibroblasts were placed in wells with 1 × 10^5^ cells per well, incubated with Dulbecco’s modified Eagle’s medium for 24 h, and then incubated with collagen peptides of different concentrations (0, 6.25, 12.5, 25.0, 50.0, 100.0, and 200 μg/mL) dissolved in culture base for 24 h, which contained 4% fetal calf serum (FCS) and 1% penicillin/ streptomycin, until confluence. The basic medium contained 4% newborn calf serum and 1% penicillin/streptomycin.

#### 2.8.1. Measurement of Cell Migration Rate

The measurement of the cell migration rate was performed as follows: 2 mL of cells at a concentration of 2 × 10^5^ cell/mL were seeded into a 6-well plate and cultured in the complete medium for 24 h. Subsequently, a sterilized 10 μL pipette tip was used to create a vertical scratch across the monolayer of cells. This scratch served as a wound model for the cultured cells. After scratching, the culture medium was aspirated, and the cells were gently washed three times with PBS to remove the detached cells; then, they were divided into three groups: a negative control group, an experimental group, and a positive control group, with three replicate wells for each group. The negative control group was cultured in the basic medium, the experimental group was cultured in the basic medium supplemented with 50 μg/mL of collagen polypeptide dissolved in the same medium, and the positive control group was cultured in the complete medium. After 12 h of incubation in the incubator, images were captured again under the inverted microscope (×100 magnification). The migration rate was calculated using the image analysis software ImageJ 1.8.0.

#### 2.8.2. Proline and Hydroxyproline Content

The content of collagen was indirectly reflected by measuring the levels of proline and hydroxyproline. Cells were seeded at a concentration of 1 × 10^5^ cell/mL in a 12-well plate with 1 mL of complete medium. After 24 h of incubation to allow cells to adhere completely, the medium in the plate was discarded, and the cells were washed twice with PBS. Negative control, experimental, and positive control groups were set up, with 3 replicate wells for each group. The negative control group was cultured in the basic medium, the experimental group was cultured in the basic medium supplemented with 50 μg/mL of collagen peptides dissolved in the same medium, and the positive control group was cultured in the complete medium. After 24 h of incubation in the incubator, the original medium was aspirated, and 0.5 mL of trypsin was added for digestion in the incubator for 1 min. Subsequently, 1 mL of the complete medium was added to terminate digestion. Following centrifugation, the supernatant was discarded, and the cells at the bottom were collected for amino acid content determination.

### 2.9. Statistical Analysis

The results are expressed as the mean ± SD. Statistical analysis was calculated by SPSS version 25.0, and one-way analysis of variance (ANOVA) was conducted. The significant differences between samples were determined at *p* < 0.05 using Duncan’s multiple range tests. The significant differences are represented by a, b, c, d, e, f, and g. Graphs were made using Origin Pro 2023 software (OriginLab Corporation, Northampton, MA, USA) and GraphPad Prism 7 (GraphPad Software Inc., San Diego, CA, USA).

## 3. Results and Discussion

### 3.1. Effect of Dual Enzyme on Hydrolysis Efficiency

#### 3.1.1. Degree of Hydrolysis

**Figure 1:**
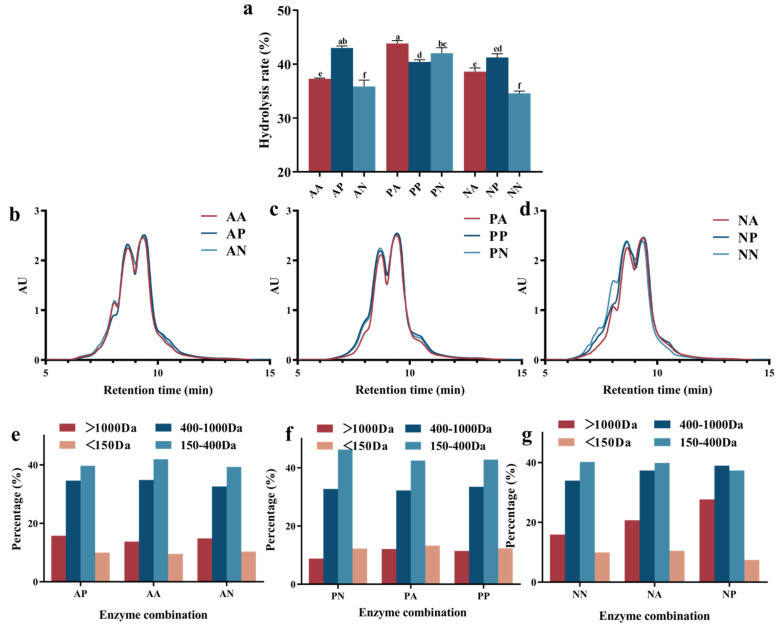
The hydrolysis rate (**a**), size exclusion chromatogram (**b**–**d**), and the calculated certain-sized collagen peptide percentage (**e**–**g**), after dual-enzyme hydrolysis. The figure b and e represented the sample treated by AA, AP, and AN. The figure c and f represented the sample treated by PA, PP, and PN. The figure d and g represented the sample treated by NN, NA, and NP.

Single-enzyme hydrolysis often exhibits some special action sites limiting the hydrolysis efficiency, whereas complex enzymes can function on more amide bonds to enhance the hydrolysis degree [14]. As shown in Figure 1a, the enzyme type and addition sequence had a strong effect on the degree of hydrolysis (DH) of fish skin collagen. In comparison, the dual enzyme had a relatively higher hydrolysis efficiency than the single enzyme, with the DH value increased significantly. The combination of alkaline and papain protease showed a higher hydrolysis efficiency than the other groups, indicating the distinct specificities of these two enzymes, contributing to the breaking of more peptide bonds and the formation of shorter-chain collagen peptides. It has been previously reported that the cumulative effect of multiple enzymes with various exogenous and endogenous activities would expose new reaction sites for other enzymes to promote further hydrolysis [15]. Furthermore, altering the enzyme addition sequence exerted a notable impact on the DH of collagen. For instance, the DH of the samples subjected to a neutral protease followed by an alkaline protease was 3% higher than that of the reverse order. This discrepancy may be related to the spatial hindrance of action sites, the addition of an enzyme with high activity first could promote the unfolding of protein molecules to expose more action sites for specific endonucleases. It could be inferred from the DH results that the optimal composite combinations are alkaline protease + papain and papain + alkaline protease, achieving DHs of 43% and 44%, respectively.

#### 3.1.2. Molecular Weight Distribution

The molecular weight distribution of collagen hydrolysates provided a more intuitive perspective on the variations in the yield of small molecular peptides [16]. Gel permeation chromatography (GPC) was employed to elucidate the patterns of molecular weight distribution under the influence of diverse enzyme combinations, with the size exclusion chromatogram shown in Figure 1b–d and the calculated certain-sized collagen peptide percentage shown in Figure 1e–g. As can be seen, the different enzyme combinations and addition sequences resulted in changes in the proportion of peptide fractions within distinct molecular weight ranges. The papain and alkaline protease hydrolyzed sample exhibited a relatively higher content of peptide fragments ranging from 400 Da to ~1000 Da and 150 Da to ~400 Da. The samples hydrolyzed by neutral protease exhibited a higher proportion of large molecular weight peptide fragments, indicating a lower hydrolytic efficiency compared to papain and alkaline protease. The segments with molecular weights less than 150 Da could be considered as free amino acids, which is more for the sample with high DH [17]. The alkaline + papain protease composite enzyme combination was selected for the subsequent processes, due to the relatively high degree of hydrolysis and the low content of free amino acids, containing approximately 43% of collagen peptides ranging from 150 Da to ~400 Da. The selected dual enzyme was employed to further investigate the effect of the triple enzyme on the hydrolysis efficiency of collagen, using ginger enzyme to hydrolyze the collagen fragments following the alkaline + papain protease composite hydrolysis.

### 3.2. Effect of Triple Enzyme on Hydrolysis Efficiency

#### 3.2.1. Degree of Hydrolysis

**Figure 2:**
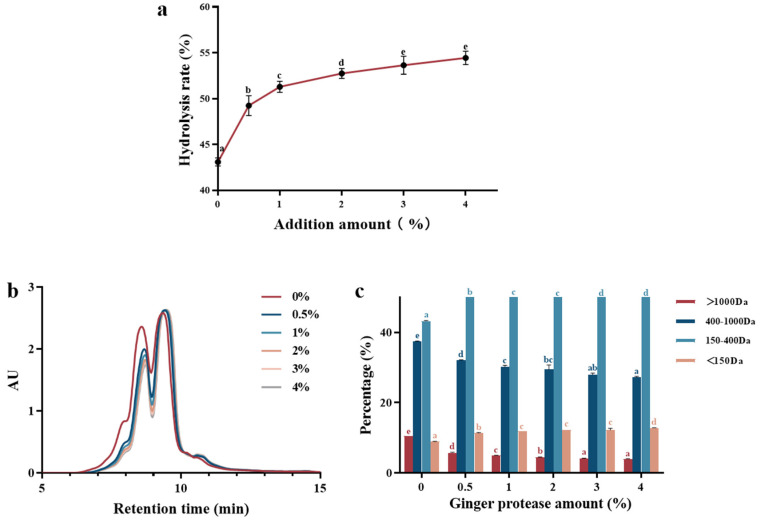
The hydrolysis degree (**a**), molecular weight (**b**), and chromatogram and molecular weight distribution (**c**) of triple-enzyme hydrolyzed collagen varied with the change in ginger protease adding amount.

To further enhance the degree of hydrolysis of collagen polypeptides, a specific ginger protease was introduced into the enzymatic digestion process. Due to the relatively high industrial cost and moderate hydrolytic capacity of ginger protease, it was necessary to employ other potent proteases initially to thoroughly disrupt the triple-helical structure of collagen before its application [18]. Figure 2a illustrates that the degree of hydrolysis of the collagen solution progressively increased with the addition of ginger protease after the dual-enzyme hydrolysis process. Specifically, when the addition level reached 0.5% (*v*/*w*), the degree of hydrolysis improved by approximately 6%, indicating the presence of residual cleavable sites in the collagen peptide residues prepared after alkaline + papain protease treatment, such as proline-rich peptides with proline C-terminal possessing high resistance to alkaline + papain protease [19]. As previously reported, proline commonly occupied the X position in the unique G-X-Y repeating sequence of collagen [6]. With regard to the specificity of ginger protease to hydrolyze the C-terminal of proline [20], it significantly contributed to the elevated degree of hydrolysis. At 3% of ginger protease addition, the degree of hydrolysis reached 54%, with little change at 4% ginger protease addition, suggesting the acting sites were completely hydrolyzed. Therefore, an enzyme addition of 3% (*v*/*w*) was used for subsequent hydrolysis, for further determination of the molecular weight distribution and peptide sequence.

#### 3.2.2. Molecular Weight Distribution

To precisely determine the effect of ginger protease hydrolysis on peptide molecular weight distribution following dual enzyme treatment, gel permeation chromatography was employed to analyze and calculate the proportions of various molecular weight peptide fractions (Figure 2b). The elution profiles of the collagen hydrolysate enabled the calculation of the percentages of different molecular weight ranges. As depicted in the Figure 2c, subsequent to ginger protease addition, the proportions of peptide fractions >1000 Da and 400 Da~1000 Da decreased, while the content of di- or tripeptides ranging among 150 Da~400 Da increased obviously. With increasing ginger protease concentrations, the molecular weight distribution shifted towards lower molecular weight fractions. This enhanced degree of hydrolysis primarily resulted from the degradation of peptides above 400 Da. The gradual increase in the content of free amino acids (<150 Da) should also be noted, suggesting that some peptides ranging from 150 Da to ~400 Da were further hydrolyzed into free amino acids. The elution profiles for the 3% and 4% addition levels nearly overlapped, confirming that enzymatic hydrolysis degree reached the limits.

### 3.3. The Sequence of Dual- or Triple-Enzyme Hydrolyzed Collagen Peptides

#### 3.3.1. Isolation of Collagen Peptides

**Figure 3:**
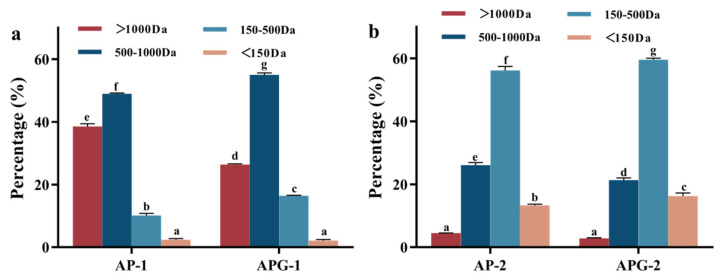
Molecular weight distribution of the samples measured via liquid chromatography: (**a**) component 1 representing the retained phase in membrane separation process and (**b**) component 2 representing the permeated phase in membrane separation process.

To further compare the activity differences between low- and high-molecular-weight peptides derived from dual-enzyme and triple-enzyme hydrolysis samples, peptides with varying molecular weight distributions were collected after 2000 Da ultrafiltration membrane filtration. The dual-enzyme hydrolyzed collagen peptides were separated into AP-1 (high-molecular-weight peptides) and AP-2 (low-molecular-weight peptides). The triple-enzyme hydrolyzed collagen peptides were segregated into APG-1 (high-molecular-weight peptides) and APG-2 (low-molecular-weight peptides). The molecular weight distribution of the collected collagen peptide fragments and the calculated percentage of peptide at various ranges are shown in Figure 3a,b. As shown in Figure 3a, in the fragment AP-1, the molecular weight primarily resides above 500 Da, accounting for 88.3% of the peptides, while the content of free amino acids below 150 Da was relatively low (<3%). Conversely, in APG-1, the proportion of peptides exceeding 500 Da slightly decreased to 81.1%. In AP-2, the molecular weight distribution shifted towards lower values, with peptides ranging from 150 Da to 500 Da comprising 55.2%, approximately four times that of AP-1. Free amino acids below 150 Da accounted for 13.6% in AP-2. Similarly, APG-2 exhibited a higher proportion of 150 Da to 500 Da peptides (59.3%), roughly three times that of AP-1, and free amino acids below 150 Da constituted 16.9% of APG-2. Molecular size is a pivotal factor influencing peptide bioavailability, as smaller peptides may be rapidly absorbed into the bloodstream and transported to tissues [21]. This aspect suggested that small molecular collagen peptides may possess enhanced biological activities. However, extensive hydrolysis also leads to an increase in free amino acids. Free amino acids could have adverse effects on the human body, such as increasing the dietary acid load in amino acid-containing medical diets, subsequently elevating urinary calcium and magnesium excretion and potentially contributing to bone fragility in patients with phenylketonuria [22]. Thus, whether AP-2 and APG-2 exhibited superior physiological activities in comparison to AP-1 and APG-1 will be subsequently investigated to validate the isolated components that exhibit superior physiological activity.

#### 3.3.2. Peptide Sequence Identification of Isolated Collagen Polypeptides

Peptidomics is a technique for the comprehensive qualitative and quantitative characterization of peptides in biological samples, primarily focused on small molecular weight proteins or peptides. Supplementary Tables S1–S4 enumerate the peptidomic identification results for four distinct collagen peptide samples. analyzing the length of peptide sequences: AP-1 was dominated by hexapeptides and heptapeptides (29 peptides); AP-2 featured an abundance of tetrapeptides and pentapeptides (126 peptides); APG-1 contained predominantly hexapeptides and heptapeptides (17 peptides), while APG-2 was rich in tetrapeptides and pentapeptides (141 peptides). This suggested that Fraction 1 primarily comprised hexapeptides and heptapeptides, whereas Fraction 2 was dominated by tetrapeptides and pentapeptides. Numerous studies have highlighted the functional value of small molecular weight peptides [23,24].

**Figure 4:**
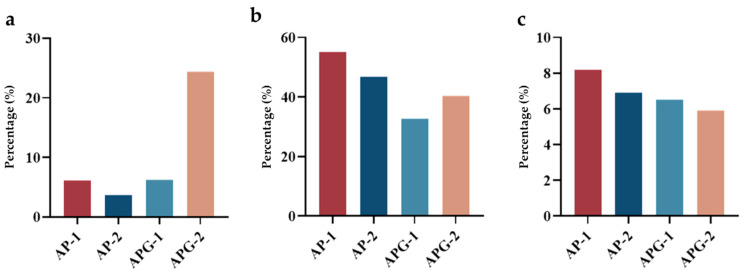
The percentage of peptide fragments with P at C- terminal (**a**), containing PO (**b**) and GPO sequences (**c**).

The number of peptides with proline terminate or containing PO and GPO sequences was calculated and compared, to intuitively compare the content of specific peptides with potential ACE inhibitory and fibroblast proliferation activity. As can be seen in Figure 4a, the number of peptides with proline terminate was different for the four fractions: AP-1 contained three such peptides, whereas AP-2 had eight, APG-1 had three, and APG-2 had fifty-four. This finding indicated that the addition of ginger protease would enhance the yield of proline-terminated peptides. Within AP-1, two peptides (AR, PGR) were confirmed to possess ACE inhibitory activity; AP-2 contained eight (AV, SV, AR, LQ, LE, RP, PR, POLG, LGL); APG-1 had nine (GR, PK, KP, AR, PR, VR, KGP, PGR, RGP); and APG-2 featured sixteen (GP, KP, RP, POGP, OGP, KGP, EQGP, WGAP, LPO, PR, VR, LR, GPAG, LGL, PGR, RR). Regarding fibroblast proliferation, Gly-Pro-Hyp and Pro-Hyp are well-studied collagen-specific sequences having the potential to promote fibroblast growth [25]. As shown in Figure 4b,c, AP-1 contained 31 peptides with GPO and PO sequences (63.3%); AP-2 had 115 (53.7%); APG-1 had 24 (39.1%); and APG-2 had 102 (46.2%). In summary, the inclusion of ginger protease led to an increase in the proline-terminated peptides but a decrease in the peptides containing PO and GPO sequences, possibly related to the cleavage of the broken of peptide bond between P and O amino acids.

### 3.4. Functional Properties of Separated Collagen Peptides

#### 3.4.1. ACE Inhibitory Activity

**Figure 5:**
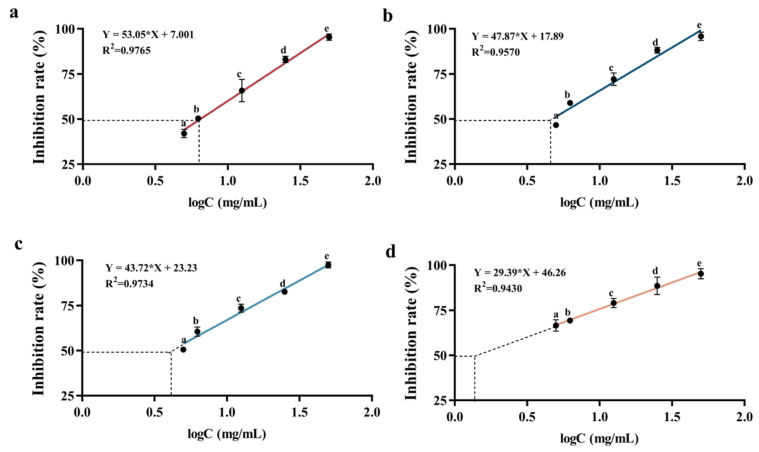
Determination of the IC50 value of the sample: (**a**) AP-1, (**b**) AP-2, (**c**) APG-1, and (**d**) APG-2. AP-1 represented the retained phase of AP dual enzyme hydrolyzed collagen peptides after membrane separation process. AP-2 represented the permeated phase of AP dual enzyme hydrolyzed collagen peptides after membrane separation process. APG-1 represented the retained phase of APG triple enzyme hydrolyzed collagen peptides after membrane separation process. AP-2 represented the permeated phase of APG triple enzyme hydrolyzed collagen peptides after membrane separation process.

The ACE inhibitory activity of samples is commonly evaluated by determining their IC50 values, which represent the concentration of peptide required to inhibit 50% of the enzyme activity and are frequently used to compare the hypotensive activity levels of hydrolysates or peptides across different studies [26]. Figure 5 illustrates the IC50 values of various collagen polypeptides, with lower values indicating higher ACE inhibitory activity. Notably, both AP-2 and APG-2 exhibited significant ACE inhibitory activity. Specifically, the IC50 value of AP-2 and APG-2 was lower than that of AP-1 and APG-2. The ACE molecule was divided into two halves by a deep and narrow channel, allowing only small peptide substrates to enter [27]. Consequently, small molecular weight peptides could readily access the ACE active site and alter its catalytic behavior, explaining the higher ACE inhibitory activity observed in the lower molecular weight fractions. Additionally, the smaller peptides may contain a higher proportion of hydrophobic amino acids such as alanine, proline, and valine, as studies have shown that peptides with higher hydrophobic values tend to exhibit lower IC50 values [28], attributed to the ACE-I enzyme preference for binding hydrophobic amino acids [10]. Molecular docking models revealed the presence of amino acid residues in ACE that formed hydrophobic pockets, encapsulating bioactive peptides, suggesting a hydrophobic interaction between bioactive peptides and ACE residues [29].

Remarkably, the IC50 value of APG-2, approximately 3.1 mg/mL, was 43.4% lower than that of AP-2, indicating that ginger protease favored the production of peptides with ACE inhibitory activity. This was attributed to ginger protease’s promotion of C-terminal proline-containing peptides, which generally exhibited high ACE inhibitory activity when proline was present at the C-terminus or in tripeptide positions. ACE possessed three primary active pockets: S1 (composed of residues Ala354, Glu384, and Tyr523), S1′ (Glu162), and S2′ (including residues Gln281, His253, Lys511, His513, and Tyr520), where inhibitors could bind [30]. Peptides terminating in proline exhibited enhanced binding affinity to these active pockets, exemplified by the formation of eight hydrogen bonds between the peptide PRP and ACE residues [31]. In conclusion, low-molecular-weight peptides possessed higher ACE inhibitory activity, and the addition of ginger protease contributed to an enhancement in this activity due to the release of proline at C-terminate and the formation of smaller sized peptides.

#### 3.4.2. Cell Migration Rate

**Figure 6:**
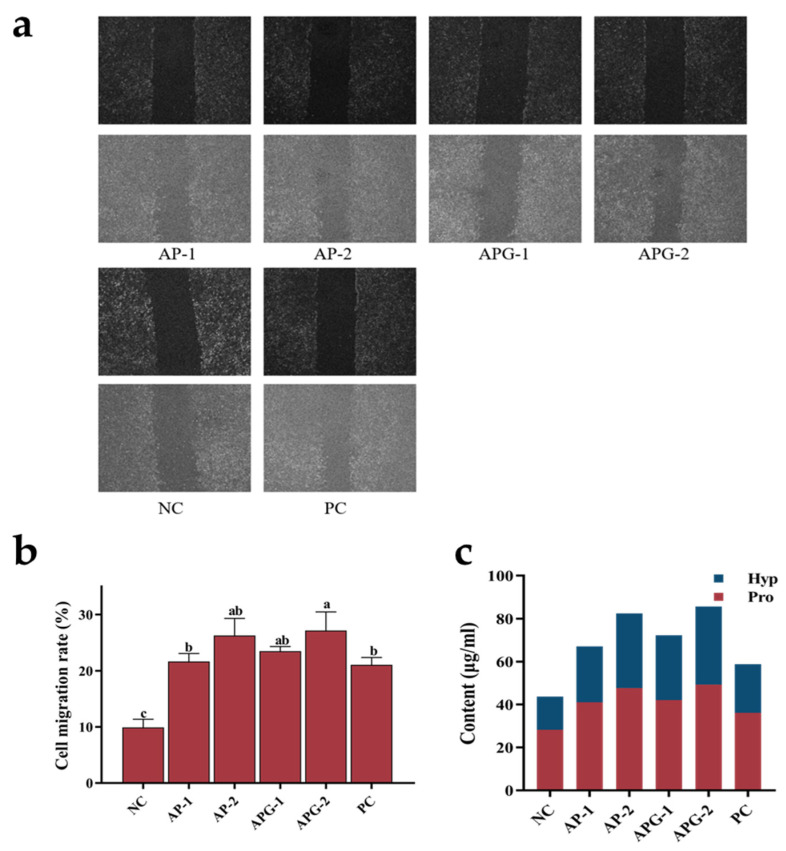
The migration (**a**), mobility (**b**), and proline/hydroxyproline contents (**c**) of fibroblasts after ingesting different samples.

Cell migration refers to the movement of cells in response to migratory signals or gradients of certain substances, playing crucial roles in numerous physiological and pathological processes, including immunity and inflammation [25]. Figure 6a depicts the migration rates of fibroblasts following exposure to collagen polypeptides of varying molecular weights, and Figure 6b presents the specific migration rates quantitatively analyzed using ImageJ software. Among the experimental groups, the cells treated with APG-2 exhibited a slightly higher migration rate than those treated with other samples, indicating the stimulatory effect of APG-2 on fibroblast migration. Notably, all sample groups displayed significantly higher migration rates than the negative control group. Furthermore, the migration rates of all sample groups were comparable to or surpassed those of the positive control group. The results indicated that the higher abundance of smaller peptides in APG-2 could activate the signaling pathways involved in promoting fibroblast migration, such as the NF-κB pathway, which was intimately linked to fibroblast migration and adhesion [32].

#### 3.4.3. Synthesizing Capability of Type I Collagen

Proline and hydroxyproline, as characteristic and abundant amino acids in collagen, serve as indicators to investigate the impact of various samples on the collagen synthesis capacity of fibroblasts by quantifying their intracellular content. Figure 6c illustrates the synthesis content of proline and hydroxyproline within fibroblasts following exposure to collagen peptides of different molecular weights. As can be seen from the figure, all the samples significantly elevated the intracellular content of both proline and hydroxyproline. Notably, APG-2 demonstrated the optimal effect in promoting the synthesis of these amino acids, indicating its potent ability to stimulate intracellular collagen production. The AP-2 group followed closely, while the AP-1 and APG-1 groups showed relatively weaker effects, yet still surpassed the positive control group. Although the peptide sequence analysis revealed a higher proportion of PO- and GPO-containing peptides in components AP-1 and APG-1, the longer peptide lengths hindered their effective degradation and utilization of the active sequences by cells. In contrast, components AP-2 and APG-2 contained predominantly shorter peptides that were more readily utilized by cells, contributing to their superior collagen synthesis capability. Additionally, other potential bioactive peptides may have influenced signal transduction pathways related to cell migration or cytoskeleton reorganization, thereby modulating the collagen synthesis capacity of cells [33]. Basically, low-molecular-weight peptides were more conducive to enhancing the synthesis of Type I collagen in fibroblasts, with a slight advantage observed in the groups treated with ginger protease.

## 4. Conclusions

Due to the specificity of ginger protease to hydrolyze the C-terminal of proline, the triple-enzyme hydrolyzed collagen peptide segments possessed a higher hydrolysis degree, containing almost 60% peptides with a molecular size smaller than 500 Da. The ginger protease addition led to an increase in proline-terminated peptides but a decrease in peptides containing PO and GPO sequences, possibly related to the cleavage of the broken peptide bonds between P and O amino acids. The formation of peptides with P at the C-terminal, and the release of more small molecular sized peptides containing hydrophobic amino acid were beneficial for the increase in ACE inhibitory activity [28]. Although the peptide sequence analysis revealed a lower proportion of PO- and GPO-containing peptides in components AP-2 and APG-2, the shorter peptide provided more action sites for effective degradation and utilization by cells, promoting fibroblast migration. Consequently, low-molecular-weight peptides were more conducive to enhancing the synthesis of Type I collagen in fibroblasts, with a slight advantage observed in the groups treated with ginger protease. The results would provide practical guidance for the development of collagen peptides with a small molecular size and high function activity.

## Data Availability

The original contributions presented in the study are included in the article/Supplementary Materials, further inquiries can be directed to the corresponding author.

## Supplementary Materials

The following supporting information can be downloaded at: https://www.mdpi.com/article/10.3390/foods13233869/s1, Table S1: Identification of the peptide sequence of AP-1; Table S2: Identification of the peptide sequence of AP-2; Table S3: Identification of the peptide sequence of APG-1; Table S4: Identification of the peptide sequence of APG-2.
