# Supplementary material for: Strategies to Improve Hydrolysis Efficiency of Fish Skin Collagen: Study on ACE Inhibitory Activity and Fibroblast Proliferation Activity
Source: Foods. 2024 Nov 29;13(23):3869. doi: 10.3390/foods13233869 (PMC11640598; doi:10.3390/foods13233869)
Supplement: Supplementary file 1 [file foods-13-03869-s001.zip › foods-3309076-supplementary.pdf]

Supplementary Table S1. Identification of the peptide sequence of AP-1.

m/z	Sequence	m/z	Sequence	m/z	Sequence
246.157	AR	557.282	PGVTAPO	571.252	GPPOGPM
301.191	PGK	557.282	VGVGPE	573.252	POGAPES
303.176	QR	557.282	PGVGDL	573.261	PVGDW
329.199	PGR	557.299	PGDRL	573.269	POGAPCL
331.214	RR	557.299	RPGEV	574.257	WSGPAG
361.177	EGR	558.247	GSPOGPQ	582.316	GPVGPVG
388.226	KGAPO	558.256	WAGPQ	585.279	PGLPOGE
414.239	KPGPO	558.256	WQGPA	586.277	QPPOGAT
414.239	POGPK	558.291	POGLQQ	587.303	POGLGLD
552.225	FGPOSE	559.214	PGEPGC	589.286	LDGDGL
552.225	PODGFT	559.266	POGQLE	610.309	GPPOGLPG
553.282	LPOGHN	561.218	POGDADA	619.266	GPPOGFE
553.29	PGLPOGP	561.227	WGEPOG	626.306	VGPPOGPS
553.299	LPGPOGP	568.281	PQGPPOG	626.315	VGPPQE
557.265	PGVGPM	568.298	GPAGPIG	629.286	PVGDPOE
557.273	PGMVGP	571.243	GPPOGNN		
557.273	VPGPMG	571.243	PGPOGNN		

Supplementary Table S2. Identification of the peptide sequence of AP-2.

m/z	Sequence	m/z	Sequence	m/z	Sequence
189.122	AV	430.19	PQGE	513.251	PGWGP
205.116	SV	430.19	PGPOGS	513.26	PGVGPS
244.128	PAG	430.197	EPGQ	515.222	PGPAAC
244.128	PGA	430.197	PGAGE	515.247	TPOGPQ
246.157	AR	430.197	PGADA	516.251	POVGGGN
260.16	LQ	430.197	DAGPA	517.214	POQGPC
261.147	LE	430.205	WPQ	517.222	QPOGAE
272.163	PGV	430.213	PGWA	517.222	QPOQE
272.169	RP	430.236	LPOGQ	517.222	POGAQE
272.175	PR	431.206	POGLE	517.23	GEAGPOA
274.178	VGV	431.214	LPOGE	525.251	PGQSH
276.157	SGL	442.273	PGAVV	525.276	PAPOGPA
301.153	GPQ	442.273	PGVGL	526.256	GPAGPQ
301.185	PKG	444.203	APOGPS	526.256	PGAGPQ
302.17	POLG	444.211	SPOGPA	527.279	PGPOAGL
302.208	LGL	446.185	QPOGE	527.279	POGPAGL
303.176	QR	446.193	POGAGE	527.287	PVSGPA
304.157	RE	454.225	POPGPA	527.313	LPOGPK
315.161	PAGA	455.23	PGGGAP	528.26	PGLPOE
315.167	PGAA	455.23	PGGGPA	529.293	POGVAVA
315.167	AGPA	458.229	DVGPA	529.293	POGVVAA
318.129	POGE	458.229	PGPTS	529.302	VPSGVA
327.171	GPPG	462.187	POGSGE	529.302	PGVSVA
327.171	PGPG	470.242	WKH	529.31	PORAVA
329.179	NVP	470.257	LPOGPA	529.31	PGVTR
329.179	GGVP	471.224	GPPOGGA	530.276	LGLPOD
329.179	PGVG	471.224	PGPOGGA	530.285	LPOGLD
329.186	GPVG	472.2	GPPOGE	530.285	LGPOLD
331.16	PGTG	472.2	PGPOGE	531.235	PGTADA
331.167	PGSA	472.272	LPOGVA	531.235	PGETGA
331.2	VGVG	474.251	POGTAL	531.252	POGERG
343.155	GPPOG	474.251	POGTLA	531.269	POGTAVA
343.196	PGAV	474.267	ERGL	535.232	POGATSS
343.196	PGLG	478.237	FPOGAA	542.25	PGSGPQ
345.171	TGPA	485.229	PGPOAQ	542.258	QPOGPQ
345.178	PGTA	485.237	APOGPQ	543.236	EPOGPQ
345.219	LGR	485.237	APOGPAG	543.236	AGPPOGE

359.192	POGAV	485.237	QPOGPA	543.236	QPPOGE
361.177	PGST	485.269	APOGPK	543.236	EGPOGPA
361.177	ERG	486.211	PGAPOE	547.295	PGAFGV
372.185	AGPQ	486.235	NGQPA	552.225	FGPOSE
372.185	PAGGA	486.251	PGALE	552.225	PODGFT
372.192	PGAAG	486.251	LPOGPS	557.265	PGVGPM
372.192	PGAGA	486.251	PGSPOL	557.273	PGMVGP
372.192	PGAQ	487.21	PGADAG	557.273	VPGPMG
372.192	QGPA	487.21	PGADQ	557.282	PGVTAPO
373.173	PGAE	487.21	PGEQG	557.282	VGVGPE
373.18	PAW	487.21	GDAGPA	557.282	PGVGDL
373.201	POLQ	487.242	SVGPQ	557.299	PGDRL
373.201	POGLA	487.251	POGAVQ	557.299	RPGEV
373.201	APOGL	487.251	GPOAGAV	558.247	GSPOGPQ
373.209	LPOQ	488.243	POGTTP	558.256	WAGPQ
388.189	POGAAG	488.243	GLPOGE	558.256	WQGPA
388.218	KGAPO	488.267	LPOGVS	558.291	POGLQQ
400.217	PGAVG	490.263	FPGAV	559.214	PGEPGC
402.201	PGTQ	490.271	PGAFV	559.266	POGQLE
405.16	EAGE	497.276	PGVGPA	561.218	POGDADA
407.179	SSPOT	499.207	PGGDPG	561.227	WGEPOG
407.179	POSST	499.249	PGTGPA	568.281	PQGPPOG
407.187	FPOGA	500.237	PGEPT	568.298	GPAGPIG
407.194	FPOQ	500.245	PVGGGN	571.243	GPPOGNN
414.232	POGPK	500.245	PVGPM	571.243	PGPOGNN
415.259	POGLL	500.253	VPGPM	571.252	GPPOGPM
415.259	LPOGL	501.234	PGPOGSA	573.252	POGAPES
416.213	PGDK	501.234	QPOGPS	573.261	PVGDW
418.206	DAGR	501.234	POGPSQ	573.269	POGAPCL
418.236	KDR	506.251	FPOGAV	582.316	GPVGPVG
428.214	PGPOAA	511.218	PGGSGH	585.279	PGLPOGE
428.214	APOGPA	511.218	PGNSH	586.277	QPPOGAT
430.167	PGGPC	511.243	GPPOGPA	587.303	POGLGLD
430.182	PGGCP	511.243	GPPGPS	619.266	PGPOGFE

Supplementary Table S3. Identification of the peptide sequence of APG-1.

m/z	Sequence	m/z	Sequence	m/z	Sequence
232.143	GR	388.226	POGAK	558.256	WAGPQ
244.168	PK	400.231	PGAR	559.24	PGAWE
244.168	KP	414.239	POGPK	561.218	POGDADA
246.157	AR	414.239	KPGPO	563.244	GEAGVM
272.175	PR	414.239	POGPK	568.272	GPPOGPQ
274.184	VR	416.228	RGPS	573.243	PVGEDG
301.185	GPK	440.223	NGLH	573.252	PEGPOTG
301.185	KGP	525.302	PGVGPV	573.261	POGAPCL
303.176	QR	551.239	SPOGFQ	573.278	POGAAEL
329.179	GPVG	552.234	PODGFT	574.249	GESGPQ
329.193	PGR	552.234	POGSFE	582.316	GPVGPVG
329.199	RGP	556.283	NAPGPT	586.277	QPGPAT
333.18	KGE	557.282	VGVGPE	589.286	LDGDGL
361.184	EGR	557.282	PGEVGV	626.278	DGPPOGPA
372.22	AGPK	557.291	PGVGDL		
388.226	KGPOA	557.308	RPGLD		

Supplementary Table S4. Identification of the peptide sequence of APG-2.

m/z	Sequence	m/z	Sequence	m/z	Sequence
173.093	GP	407.194	FPOQ	486.251	POGPSL
244.128	PGA	414.194	GAPOGP	487.21	PAGDAG
244.168	KP	414.194	GPSGP	487.21	PAGDQ

245.153	LPO	414.194	PGSGP	487.21	PGADAG
260.124	PGS	414.194	PGPOQ	487.21	PGDAGA
260.124	POGA	414.202	QPPOG	487.21	PGDGAA
272.163	PGV	414.202	GPOPGA	488.227	POGLGE
272.169	RP	414.202	GPPOGA	490.23	GFPOGP
272.169	PR	414.232	PGAAV	490.238	POGFPG
274.19	VR	415.222	LPGE	495.249	GPPGPA
276.157	SGL	415.259	LSVP	497.268	PGVGPA
286.135	POGP	415.259	POLGL	499.216	DEVH
288.2	LR	424.215	GPPGP	499.249	GPAGPT
301.146	GPAG	426.204	NGVH	501.225	PGPOGSA
301.153	QGP	426.219	RGGH	501.225	PPOGSQ
301.185	PGK	426.227	GPVGP	501.234	QEGAP
301.191	KGP	426.227	PGVGP	501.234	GPPOGSA
302.208	LGL	428.206	POAGAP	501.234	QPOGPS
303.163	VGQ	428.214	APOGPA	501.234	PGSGPS
303.169	QGV	430.174	GPGPC	506.259	POFGAV
315.167	PAGA	430.19	EQGP	506.267	POGFAV
315.167	PGAA	430.19	POGSGP	506.267	FPOGAV
317.151	POGQ	430.19	GPAGE	511.243	AGPPOGP
318.129	POGE	430.197	DAGAP	511.243	GPPGPS
327.171	GPPG	430.197	PQGE	511.251	QPPOGP
327.171	PGPG	430.197	EGPQ	511.251	GPPGPOA
329.179	GPVG	430.197	PGEQ	511.284	PGIGPA
329.193	PGR	430.205	WGAP	513.251	PGWGP
331.16	PGTG	430.205	WPQ	513.26	PGVGPS
331.167	PGSA	430.205	PGWA	513.268	PGVGSP
331.214	RR	431.214	LPOGE	515.222	PGAPAC
331.214	VGR	433.197	MWP	515.239	TPOGPQ
341.187	APGP	433.212	FPOGP	515.239	VPGADG
343.155	GPPOG	440.192	NGPOH	516.251	VPOGPM
343.196	PGLG	440.207	GPPOGP	517.23	POQGAE
345.171	PGTA	440.254	PGIGP	525.26	PSPGPA
345.178	TGPA	442.265	PGVGL	525.276	PGKSH
345.219	LGR	442.273	PGVAV	525.293	GPVGPV
357.171	APOGP	444.203	APOGPS	525.319	PRGPV
361.177	EGR	444.211	PSGPOA	528.26	PGLPOE
361.177	RGE	444.219	PGDR	528.269	PGLQN
361.177	PGST	446.193	POGAAD	529.302	VAPOGVA
372.185	PAGGA	454.233	PGPPOA	530.276	LPOGLD
372.185	PGQA	454.233	PPOGPA	531.235	POGADGV
372.192	QGAP	455.222	GPQGP	531.269	POGTAVA
372.192	AGPQ	455.222	PGQGP	531.278	SVPGTA
372.192	PGAGA	455.222	PQGGP	533.232	PSGSW
372.22	KPGA	455.222	NPAGP	539.278	VGPPOGP
373.166	AGPE	458.205	PGMGP	542.25	QPOGPQ
373.173	SPOGP	458.205	GPMGP	543.236	EGPOGPA
373.173	PGAE	458.276	POGLR	543.245	PGSGEP
373.201	POGLA	460.202	POAGAE	543.245	PEGSGP
373.201	POLQ	462.179	POGSGE	543.245	PGESGP
373.201	LPOQ	463.256	FSLP	543.245	PGSGPE
373.201	APOGL	470.226	PEGAP	551.239	SPOGFQ
384.187	GPPGG	470.257	WKH	552.234	PODGFT
384.187	PGPGG	470.257	LPOGPA	552.234	POGSFE
385.249	PGLV	471.216	GPPOGQ	556.283	NAPGPT
385.249	PGVL	471.224	PGPOGQ	557.282	VGVGPE
386.204	AAGAP	471.224	GPPOGGA	557.282	PGEVGV
386.211	PGGR	472.2	GPPOGE	557.291	PGVGDL
387.181	TPOGP	472.28	IGSVP	557.308	RPGLD
388.182	PGSQ	472.28	POGVIA	558.256	WAGPQ
